# Hysteroscopic Diagnosis of a Concurrent Placental Site Nodule and Cervical Adenocarcinoma: A Case Report

**DOI:** 10.7759/cureus.98892

**Published:** 2025-12-10

**Authors:** Mai Yamazoe, Yosuke Tarumi, Sakura Yamamoto, Yoichiro Fujiwara, Taisuke Mori, Akemi Koshiba

**Affiliations:** 1 Obstetrics and Gynaecology, Kyoto City Hospital, Kyoto, JPN; 2 Obstetrics and Gynaecology, Kyoto Prefectural University of Medicine, Kyoto, JPN

**Keywords:** case report, cervical adenocarcinoma, hysteroscopy, placental site nodule (psn), uterine cervical cancer

## Abstract

A 32-year-old woman presented with atypical glandular cells on cervical cytology. Her obstetric history was gravida 2 para 2, with 23 months since her last pregnancy. Colposcopy and biopsy revealed cervical adenocarcinoma. Magnetic resonance imaging and positron emission tomography/computed tomography showed no distant spread but suggested a uterine lesion, which was suspected to be corpus uterine cancer. Hysteroscopy revealed a 15-mm white nodular lesion in the lower uterine body; biopsy suggested a placental site nodule (PSN). Cervical conization confirmed stage IA2 cervical adenocarcinoma. The patient underwent a radical hysterectomy. Final pathology showed no residual carcinoma. The uterine lesion was confirmed as benign PSN via immunohistochemistry. She remained recurrence-free at the two-year follow-up. This is the first reported case of concurrent PSN and cervical adenocarcinoma. Visualization and targeted biopsy allowed accurate diagnosis of PSN, preventing overtreatment. The case highlights the diagnostic value of hysteroscopy and the importance of histological evaluation to differentiate PSN from malignancy.

## Introduction

Placental site nodule (PSN) is a rare, benign, non-neoplastic nodular lesion caused by abnormal proliferation of intermediate trophoblasts from a previous pregnancy in women of childbearing age [1]. PSN results from incomplete involution of the implantation site within the endometrium [2,3]. It is asymptomatic from weeks to years postpartum and often found incidentally during cervical cytology, cervical curettage, or infertility evaluation; however, it may also be detected following presentation of irregular genital bleeding or amenorrhea [2]. PSN occurs very infrequently, and its prevalence in the general population is unknown; however, recent research indicates that it may contribute to infertility and recurrent pregnancy loss [4]. As PSN occurs inside the uterus, it is important to diagnose its presence appropriately and, in some cases, distinguish it from malignant lesions.

Herein, we report a case in which PSN coexisted with cervical adenocarcinoma in a patient. Despite a difficult diagnostic process, hysteroscopic biopsy enabled a preoperative diagnosis of PSN, allowing appropriate treatment for the concurrent cervical malignancy.

## Case presentation

**Figure 1:**
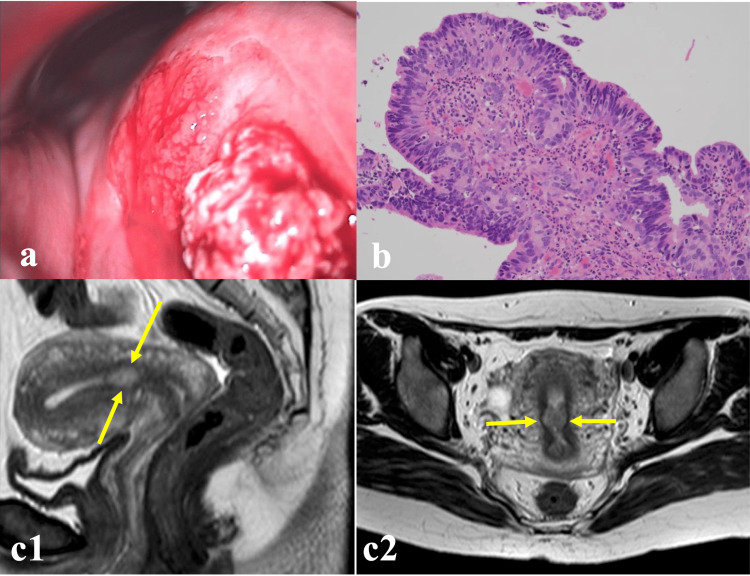
Findings of colposcopy, biopsy, and MRI (a) Colposcopy showing a pea-sized mastoid-like lesion at 2 o'clock on the cervix, and an erosive lesion with an irregular surface at 11 o'clock. (b) Adenocarcinoma, with a finding of a histologically highly columnar epithelium with prominent nuclear atypia, hematoxylin and eosin (H&E) staining, ×40. (c1) (c2) Sagittal plane (c1), and transverse plane (c2) MRI T2W1 showing circumferential thickening (yellow arrows) of the endometrium in the uterine body near the inner uterine orifice.

A 32-year-old Japanese woman, gravida 2 para 2 (two normal vaginal deliveries), with a BMI of 25.6 kg/m^2^, presented to our hospital following the detection of atypical glandular cells through cervical cytology at her annual Pap screening during a standard checkup. She had delivered her second normal full-term infant 23 months prior to the initial visit to our hospital. Her menstrual period was a regular cycle every 40 days, with no history of irregular bleeding. She had no chief complaints, past medical history, surgical history, family history, or medication history. Blood testing revealed no obvious abnormalities. Colposcopy revealed a pea-sized mastoid-like lesion at 2 o'clock on the cervix, and an erosive lesion with an irregular surface at 11 o'clock (Figure 1a). Punch biopsies were taken from both sites; pathological examination of the 11 o'clock lesion revealed adenocarcinoma (Figure 1b). Magnetic resonance imaging (MRI) revealed a circumferential thickening of the endometrium (T2WI moderately high) in the uterine body near the inner uterine orifice. The cervical lesion was not clearly visible on MRI; however, the junctional zone was preserved, and no clear muscle layer invasion, ascites, or significant lymphadenopathies were observed (Figures 1c1-1c2). Positron emission tomography-computed tomography (PET-CT) was performed to evaluate metastasis and showed no significant accumulation in the cervix, no mass suspicious for metastasis, and no abnormal fluorodeoxyglucose (FDG) accumulation. Based on the MRI findings, we considered the possibility of synchronous cervical and endometrial carcinoma.

**Figure 2:**
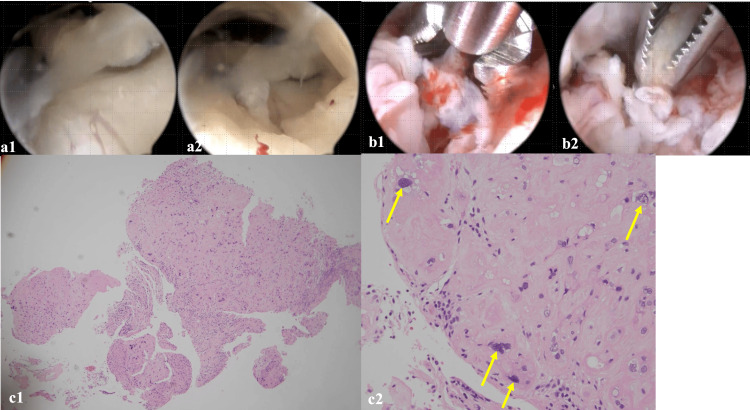
Hysteroscopy and H&E findings of a placental site nodule (PSN) (a1) (a2) Hysteroscopy, showing a white polyp. (b1) (b2) Hysteroscopic sampling with scissors, surrounded by endometrium prone to bleeding. (c1) (c2) Pathological findings after hysteroscopy showing a placental site nodule, with multinucleated cells (yellow arrow) and a vitreous-like nodular lesion in the surrounding area. Cell density is low, and no mitotic figures are observed. H&E staining, ×40 (c1), ×200 (c2).

To further evaluate the uterine lesion, we performed a hysteroscopy and biopsy, followed by endometrial curettage, and cervical conization for cervical adenocarcinoma staging while the patient remained under anesthesia. Using BETTOCHI® rigid hysteroscopy (KARL STORZ SE & Co. KG, Tuttlingen, Germany), we inserted the hysteroscope into the uterine canal via the cervix. Fine-diameter rigid hysteroscopy revealed a white nodular lesion in the lower uterine body. The lesion was 15 mm in diameter, with an irregular surface around the circumference, areas prone to bleeding in the proximity, and increased vascularization (Figures 2a1-2a2). A portion of the nodule was excised using 5-Fr scissors forceps for pathological examination (Figures 2b1-2b2). Hematoxylin and eosin-stained pathological examination of the excisional biopsy in the vision of hysteroscopy revealed that the lesion had multinucleated cells, while a vitreous-like nodular lesion was observed in the surrounding area (Figures 2c1-2c2), raising suspicion of a placental site nodule. Adenocarcinoma showing papillary structures was detected through endometrial and cervical curettage, which was considered contamination of the cervical lesion. HPV-associated adenocarcinoma pT1a2 (depth of invasion: 3.2 mm; maximal longitudinal length: 7.5 mm) was detected from the conical excised lesion. At that time, the patient was diagnosed with a cervical adenocarcinoma (FIGO 2018 IA2 stage, pT1a2 cN0M0).

**Figure 3:**
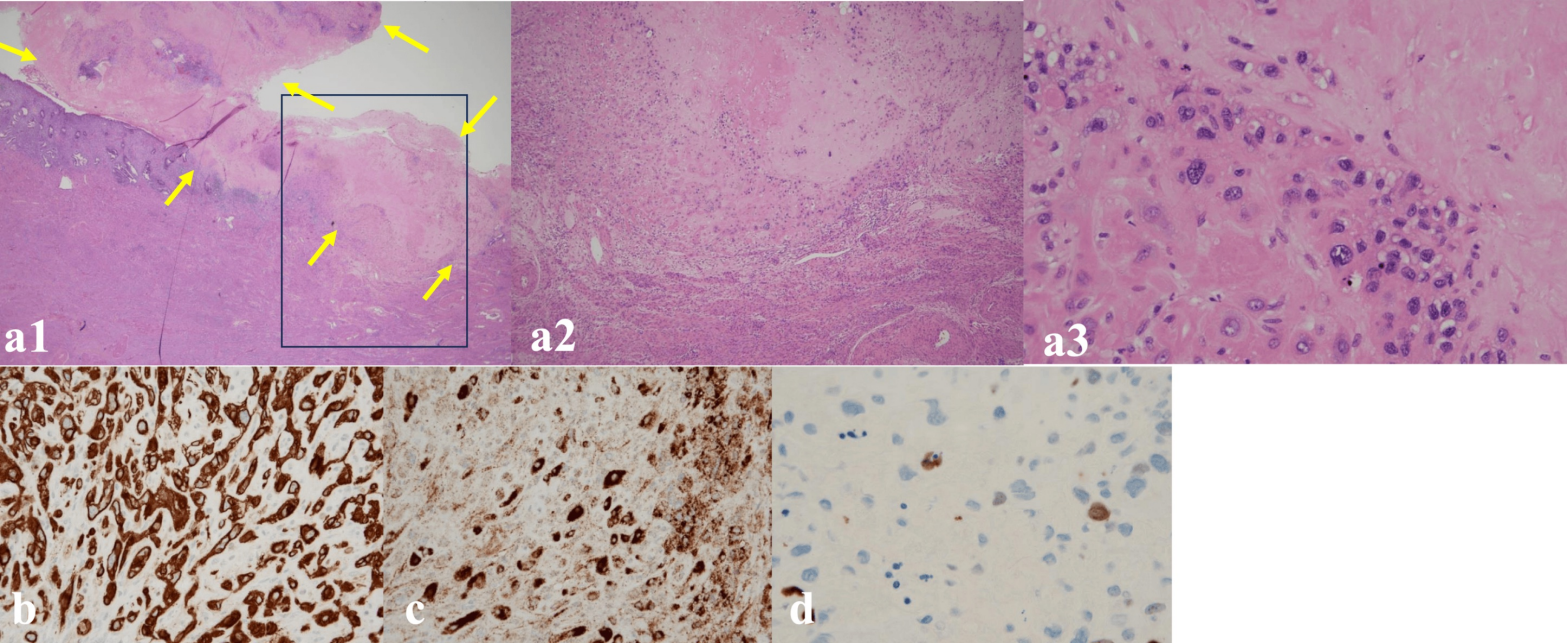
Pathological findings of hysterectomy specimens (a1)-(a3) Pathological findings after radical hysterectomy showing circumscribed, paucicellular hyalinized nodules composed of intermediate trophoblast, and placental site nodule (yellow arrow, 15 mm), ×40 (a2), ×200 (a3). There is no obvious myometrial invasion. Partial magnification of the stalked nodule lesion reveals a vitreous-like nodule lesion with multinucleated cells. (b)-(d) Low-grade malignancy with positive AE1+AE3, inhibin α, and Ki-76<5%. (b) Immunostaining for AE1/AE3, pan-epithelial marker, showing diffuse positivity, ×200. (c) Immunostaining for inhibin showing positive staining trophoblastic cells in the placental nodule, inhibin immunostaining, ×200. (d) Immunostaining for Ki67, cell division marker, less than 5％, Ki-67 immunostaining, ×400.

Consequently, we performed a radical hysterectomy. Intraoperative intra-abdominal findings showed no abnormalities, and cytology of the ascites returned negative results. The final histopathological finding revealed no residual adenocarcinoma in the cervix and no malignancies in the bilateral adnexa and pelvic lymph nodes (0/41). Therefore, the patient was finally classified as FIGO2018 IA2 stage and required no adjuvant therapy. The nodular lesion in the lower uterine body was ultimately diagnosed as a placental site nodule (Figures 3a1-3a3). There was no obvious myometrial invasion. Further immunostaining of this vitreous-like nodular lesion revealed a low grade of malignancy positive for AE1+AE3 (Figure 3b), inhibin α (Figure 3c), and Ki-76<5% (Figure 3d). 

The patient was discharged on the eighth postoperative day without any complications. She is currently undergoing outpatient follow-up and has shown no signs of recurrence two years after the last surgery.

## Discussion

To the best of our knowledge, this is the first reported case of coexisting PSN and cervical adenocarcinoma. This is a very rare condition, and the diagnosis based solely on conventional colposcopy, biopsy, and imaging diagnosis using MRI was difficult. Nonetheless, the use of a fine, rigid hysteroscopic-targeted biopsy enabled an accurate preoperative diagnosis.

Data regarding how PSNs affect women of reproductive age are scarce, despite the fact that the average age at diagnosis of PSN is 31.1 years, with cases reported in adults between 20 and 47 years of age. The interval from the latest pregnancy to detection ranges from one month to nine years, with a mean of three years. Previous reports have indicated that 45-82% of patients with PSN have a history of dilation and curettage or cesarean section delivery prior to their most recent pregnancy [2,3,5].

PSN can occur anywhere in the uterine cavity, but can be difficult to diagnose when found in atypical sites such as the cervix or the lower-uterine segment or fallopian tubes [6-8], or when incidentally coexisting with other potentially malignant lesions. In prior case series, a notable proportion (33-69%) of PSN lesions developed in the lower uterine segment [2,3,9], which is the same region as in our case.

Typically, PSN involves benign nodules or plaques, usually <5 mm in size. Microscopic examination of these lesions generally reveals a well-defined, rounded lesion consisting of subinvoluted vessels, intermediate trophoblasts, and abundant extracellular hyalinized material [5]. PSNs are characterized by chorionic-type intermediate trophoblasts arranged as single cells or cords with an abundant hyalinized matrix. Typical histological features include focal cytological atypia, enlarged hyperchromatic nuclei with smudgy chromatin, and absent or minimal mitotic activity. The Ki-67 proliferation index is <5%. Although PSN is classified as a benign gestational trophoblastic disease, atypical PSN (aPSN: higher cell count, nuclear atypia, mitotic activity, and Ki-67 proliferation index compared to PSN) has been suggested as being associated with trophoblastic malignancy [10,11]. PSNs may also be histologically confused with squamous cell carcinoma of the cervix; however, PSNs are immunoreactive for inhibin-α and cytokeratin-18, whereas squamous cell carcinomas are negative [5]. Few reports exist on how to distinguish cervical squamous cell carcinoma and PSN. Indeed, this is the first report of the incidental coexistence of PSN and cervical adenocarcinoma in a previously healthy, asymptomatic woman. We found no evidence to suggest any pathological associations between PSN and cervical adenocarcinoma; however, based on the pathogenesis of PSN, we hypothesized that the lesions arose independently.

A specific treatment with long-term follow-up is unnecessary for PSN [3,5]. Further, in cases of potentially malignant uterine lesions with a similar presentation, timely diagnosis and treatment are necessary to differentiate them from PSN. In the present case, we first performed hysteroscopy and biopsy, followed by endometrial curettage and cervical conization. No clear rule has been established as to whether conical resection should precede endometrial curettage, and whether endometrial curettage or conical resection should be performed first remains debatable. We weighed the associated risks of contamination of the lesion and increased bleeding and chose to perform exploration of the uterine cavity first. However, identifying whether the adenocarcinoma detected by endometrial curettage was a contamination of the cervical lesion or was initially detected from a uterine body lesion proved difficult. Using a fine-diameter rigid hysteroscope, we were able to confirm the location of the lesion and directly obtain a specimen to determine that the adenocarcinoma detected by the endometrial and cervical curettage was a contamination of the cervical lesion. The choice of which procedure is performed first is case dependent; however, the present case indicates that hysteroscopy should be considered in cases where the lesions need to be visualized directly.

A few case reports have noted a variety of hysteroscopic findings with PSN [12]. One case in 1994 described a 2 cm irregular-surfaced white-red nodule with regions of hemorrhage, necrosis, and increased vascularity in nearby regions [9]; another in 2005 reported an intrauterine adhesion with a small yellow-white necrotic nodule below it [13].

## Conclusions

In conclusion, we present a rare case of PSN coexisting with cervical adenocarcinoma. Since curettage is a blind procedure, this case demonstrates that hysteroscopy, which allows direct observation and sampling of the lesion under actual specular vision and tissue sampling, is beneficial for the diagnosis of endometrial lesions that are concurrent with cervical cancer.
